# The Concentration of Trace Elements in Human Lithogenic Bile

**DOI:** 10.1155/1996/26323

**Published:** 1996

**Authors:** Mohamad Abid Bakhotmah

**Affiliations:** University Hospital Department of Surgery P.O. Box 6615 Jeddah 21452 Saudi Arabia


*HPB Surgery*, 1996, Vol.9, pp.161–164
Reprints available directly from the publisher
Photocopying permitted by license only

© 1996 OPA (Overseas Publishers Association)
Amsterdam B.V. Published in The Netherlands
by Harwood Academic Publishers GmbH
Printed in Malaysia

### The Concentration of Trace Elements in Human Lithogenic Bile

MOHAMAD ABID BAKHOTMAH

[1]Consultant Hepatobiliary Surgeon and Assistant Professor

*(Received 12 October 1993)*

### INTRODUCTION

The trace element content of human bile was studied to find the difference between hepatic, common bile duct and gallbladder bile. Concentrations of trace elements in gallbladder bile is more than that of hepatic bile and this observation can not be explained as due to simple water absorption. The zinc is exceptional in that, it is 3.6 times lower in gallbladder bile than in hepatic bile.

### METHODS

Bile samples were collected from 43 patients with gallstones. Bile from PTC tubes was used for hepatic bile, bile aspirated from the common bile duct was used for common bile duct bile; and bile aspirated from the gallbladder was when as gallbladder bile.

Immediately, after collection, the sample was stored at –70°C; on the day of analysis, all samples were thawed at room temperature. Analytical grade hydrochloric acid (A.R.) Hcl was diluted by deionized water to three molar (3M). Every sample was then treated individually as follows: one minute hand shaking; 10 ml. of bile was aspirated and placed in a 250 ml beaker, 50 ml of the 3M HCl was then added. All specimens were then kept in a boiling water bath for 30 minutes followed by 5 minutes on a burner, to ensure adequate digestion. After filtration, the resultant clear solution was made upto 100ml in a volumetric flask by adding deionized water.

The content of the elements in the 3M HCl (Blank) was determined first (for the calibration curve) and if any elements existed thus was subtracted from the value for the bile solution. This figure is then taken as the concentration of the elements in bile. For measuring the (Aluminium (Al), Barium (Ba), Calcium (Ca), Molybdenum (Mo), Phosphorous (P), Silicon (Si), Titanium (Ti), Vanadium (V) and the inductively coupled plasma (ICP) Model IL phase 200 was used; for measuring the (Sodium (Na), Potassium (K), Nichel (Ni), Manganese (Mn), Lead (Pb), Silver (Ag), Calcium (Ca) Zinc (Z), Cobolt (Co), Chromium (Ch), Copper (Cu), Iron (Fe) atomic absorption spectrophotometry (A.A.S) was used. The concentrations are expressed by part per million (P.P.M.) i.e. μg/ml. Computing the results was accomplished by the SPSSPC$^+$ program.

### RESULTS

The different concentrations of elements in the bile are shown in (Table 1). The mean values are shown in Table 2.

### DISCUSSION

To my knowledge, the use of a chemical digestion method, and a Comparison of hepatic and gallbladder bile has not been previously, carried out. The chemical

*Correspondence to:* M.A. Bakhotmah, University Hospital, Department of Surgery, P.O. Box 6615, Jeddah 21452, Saudi Arabia

**Table 1** Trace Element Content of Human Lithogenic Bile In all 43 Analyzed Samples

| *Na* | *K* | *Mg* | *Ni* | *Mn* | *Pb* | *Ag* | *Cd* | *Zn* | *Co* | *Cr* | *Cu* | *Fe* | *Al* | *Ba* | *Ca* | *Mo* | *P* | *Si* | *SrO* | *Ti* | *V* |
|---|---|---|---|---|---|---|---|---|---|---|---|---|---|---|---|---|---|---|---|---|---|
| 287.50 | 60.80 | 4.78 | 0.00 | 0.190 | 0.831 | 0.000 | 0 | 0.662 | 0 | 0 | 0.316 | 0.763 | 0 | 0 | 7.51 | 0 | 18.83 | 0 | 0 | 0 | 0 |
| 322.00 | 53.70 | 13.95 | 0.00 | 0.278 | 0.432 | 0.000 | 0 | 0.166 | 0 | 0 | 0.943 | 0.735 | 0 | 0 | 25.68 | 0 | 119.35 | 0 | 0 | 0 | 0 |
| 483.00 | 38.97 | 4.99 | 0.00 | 0.124 | 0.348 | 0.000 | 0 | 0.246 | 0 | 0 | 0.244 | 0.330 | 0 | 0 | 11.50 | 0 | 36.19 | 0 | 0 | 0 | 0 |
| 586.00 | 60.30 | 14.47 | 0.00 | 0.273 | 0.642 | 0.360 | 0 | 0.251 | 0 | 0 | 0.797 | 1.000 | 0 | 0 | 30.99 | 0 | 104.71 | 0 | 0 | 0 | 0 |
| 260.00 | 43.70 | 10.28 | 0.00 | 0.340 | 0.827 | 0.194 | 0 | 0.601 | 0 | 0 | 0.692 | 0.883 | 0 | 0 | 17.46 | 0 | 74.02 | 0 | 0 | 0 | 0 |
| 550.00 | 80.00 | 26.87 | 0.00 | 0.314 | 0.610 | 0.126 | 0 | 0.300 | 0 | 0 | 2.541 | 1.940 | 0 | 0 | 63.72 | 0 | 190.49 | 0 | 0 | 0 | 0 |
| 468.50 | 76.00 | 29.33 | 0.00 | 0.225 | 0.662 | 0.073 | 0 | 0.251 | 0 | 0 | 1.554 | 0.883 | 0 | 0 | 62.51 | 0 | 239.48 | 0 | 0 | 0 | 0 |
| 374.00 | 49.42 | 1.84 | 0.00 | 0.055 | 1.200 | 0.000 | 0 | 0.353 | 0 | 0 | 0.396 | 0.583 | 0 | 0 | 67.25 | 0 | 2.45 | 0 | 0 | 0 | 0 |
| 350.50 | 45.30 | 4.43 | 0.00 | 0.101 | 0.243 | 0.000 | 0 | 0.410 | 0 | 0 | 0.490 | 0.834 | 0 | 0 | 15.20 | 0 | 4.60 | 0 | 0 | 0 | 0 |
| 473.00 | 64.00 | 12.60 | 0.00 | 0.172 | 0.300 | 0.109 | 0 | 0.210 | 0 | 0 | 0.747 | 0.804 | 0 | 0 | 30.67 | 0 | 113.10 | 0 | 0 | 0 | 0 |
| 398.00 | 54.40 | 2.55 | 0.00 | 0.200 | 0.250 | 0.050 | 0 | 1.665 | 0 | 0 | 0.587 | 0.492 | 0 | 0 | 14.67 | 0 | 7.40 | 0 | 0 | 0 | 0 |
| 272.00 | 42.40 | 7.90 | 0.00 | 0.089 | 0.000 | 0.000 | 0 | 0.534 | 0 | 0 | 1.179 | 0.400 | 0 | 0 | 18.27 | 0 | 40.00 | 0 | 0 | 0 | 0 |
| 445.00 | 75.60 | 8.80 | 0.00 | 0.158 | 0.367 | 0.000 | 0 | 0.474 | 0 | 0 | 0.616 | 0.390 | 0 | 0 | 30.81 | 0 | 74.42 | 0 | 0 | 0 | 0 |
| 404.00 | 34.50 | 4.61 | 0.00 | 0.000 | 0.374 | 0.000 | 0 | 0.162 | 0 | 0 | 0.435 | 0.220 | 0 | 0 | 13.40 | 0 | 23.31 | 0 | 0 | 0 | 0 |
| 561.00 | 78.00 | 22.20 | 0.00 | 0.297 | 0.442 | 0.000 | 0 | 0.184 | 0 | 0 | 1.102 | 0.530 | 0 | 0 | 57.66 | 0 | 265.11 | 0 | 0 | 0 | 0 |
| 570.00 | 107.25 | 25.00 | 0.00 | 0.237 | 0.827 | 0.000 | 0 | 0.600 | 0 | 0 | 1.205 | 2.605 | 0 | 0 | 57.54 | 0 | 190.46 | 0 | 0 | 0 | 0 |
| 569.00 | 66.70 | 17.51 | 0.13 | 0.134 | 0.335 | 0.000 | 0 | 0.204 | 0 | 0 | 1.362 | 0.543 | 0 | 0 | 53.56 | 0 | 189.25 | 0 | 0 | 0 | 0 |
| 673.50 | 109.30 | 33.80 | 0.00 | 0.645 | 0.360 | 0.220 | 0 | 0.300 | 0 | 0 | 1.880 | 1.010 | 0 | 0 | 99.37 | 0 | 315.00 | 0 | 0 | 0 | 0 |
| 564.50 | 112.90 | 17.76 | 0.00 | 0.378 | 0.340 | 0.050 | 0 | 0.355 | 0 | 0 | 0.844 | 1.365 | 0 | 0 | 46.30 | 0 | 173.00 | 0 | 0 | 0 | 0 |
| 504.50 | 48.86 | 4.47 | 0.00 | 0.218 | 0.301 | 0.000 | 0 | 0.163 | 0 | 0 | 0.441 | 0.440 | 0 | 0 | 11.67 | 0 | 38.34 | 0 | 0 | 0 | 0 |
| 479.00 | 23.54 | 2.12 | 0.00 | 0.239 | 0.480 | 0.000 | 0 | 0.262 | 0 | 0 | 0.471 | 0.400 | 0 | 0 | 11.27 | 0 | 4.58 | 0 | 0 | 0 | 0 |
| 585.00 | 75.30 | 13.77 | 0.00 | 0.493 | 0.000 | 0.000 | 0 | 0.191 | 0 | 0 | 1.267 | 0.620 | 0 | 0 | 28.97 | 0 | 158.30 | 0 | 0 | 0 | 0 |
| 422.50 | 52.20 | 8.30 | 0.00 | 0.240 | 0.000 | 0.000 | 0 | 0.270 | 0 | 0 | 2.396 | 0.571 | 0 | 0 | 30.81 | 0 | 74.42 | 0 | 0 | 0 | 0 |
| 423.00 | 50.60 | 3.36 | 0.00 | 0.344 | 0.410 | 0.000 | 0 | 0.200 | 0 | 0 | 0.451 | 0.550 | 0 | 0 | 11.11 | 0 | 15.76 | 0 | 0 | 0 | 0 |
| 850.50 | 76.10 | 3.45 | 0.00 | 0.100 | 0.300 | 0.000 | 0 | 0.227 | 0 | 0 | 0.399 | 0.486 | 0 | 0 | 20.37 | 0 | 2.68 | 0 | 0 | 0 | 0 |
| 435.50 | 45.38 | 2.85 | 0.00 | 0.050 | 0.440 | 0.000 | 0 | 0.500 | 0 | 0 | 0.468 | 0.500 | 0 | 0 | 12.43 | 0 | 9.61 | 0 | 0 | 0 | 0 |
| 655.00 | 90.00 | 18.00 | 0.00 | 0.385 | 0.270 | 0.000 | 0 | 0.300 | 0 | 0 | 0.563 | 0.970 | 0 | 0 | 40.76 | 0 | 275.90 | 0 | 0 | 0 | 0 |
| 700.00 | 115.20 | 26.55 | 0.00 | 0.971 | 0.240 | 0.000 | 0 | 0.310 | 0 | 0 | 0.560 | 1.400 | 0 | 0 | 88.45 | 0 | 236.20 | 0 | 0 | 0 | 0 |
| 550.25 | 100.00 | 14.41 | 0.00 | 0.345 | 0.280 | 0.000 | 0 | 0.600 | 0 | 0 | 0.770 | 1.500 | 0 | 0 | 41.52 | 0 | 155.50 | 0 | 0 | 0 | 0 |
| 784.50 | 117.90 | 27.30 | 0.00 | 0.610 | 0.220 | 0.000 | 0 | 0.220 | 0 | 0 | 1.319 | 1.420 | 0 | 0 | 85.58 | 0 | 174.70 | 0 | 0 | 0 | 0 |
| 698.50 | 90.00 | 29.40 | 0.00 | 0.070 | 0.290 | 0.000 | 0 | 0.253 | 0 | 0 | 1.297 | 0.731 | 0 | 0 | 64.62 | 0 | 222.30 | 0 | 0 | 0 | 0 |
| 550.00 | 60.50 | 7.50 | 0.00 | 0.152 | 0.200 | 0.000 | 0 | 0.172 | 0 | 0 | 0.469 | 1.193 | 0 | 0 | 17.14 | 0 | 55.28 | 0 | 0 | 0 | 0 |
| 350.00 | 40.50 | 11.13 | 0.00 | 0.384 | 0.000 | 0.000 | 0 | 0.321 | 0 | 0 | 0.718 | 2.280 | 0 | 0 | 57.27 | 0 | 62.62 | 0 | 0 | 0 | 0 |
| 507.00 | 44.40 | 3.24 | 0.00 | 0.093 | 1.010 | 0.000 | 0 | 0.175 | 0 | 0 | 0.406 | 0.374 | 0 | 0 | 10.37 | 0 | 10.81 | 0 | 0 | 0 | 0 |
| 305.00 | 34.00 | 1.49 | 0.00 | 0.050 | 0.000 | 0.000 | 0 | 0.170 | 0 | 0 | 0.443 | 0.500 | 0 | 0 | 6.81 | 0 | 2.95 | 0 | 0 | 0 | 0 |
| 542.00 | 48.30 | 5.82 | 0.00 | 0.062 | 0.000 | 0.000 | 0 | 0.882 | 0 | 0 | 0.669 | 0.520 | 0 | 0 | 19.86 | 0 | 41.55 | 0 | 0 | 0 | 0 |
| 483.50 | 44.00 | 5.56 | 0.00 | 0.071 | 0.000 | 0.000 | 0 | 0.244 | 0 | 0 | 0.448 | 0.600 | 0 | 0 | 15.33 | 0 | 36.50 | 0 | 0 | 0 | 0 |
| 510.00 | 52.60 | 5.21 | 0.00 | 0.104 | 0.000 | 0.000 | 0 | 0.147 | 0 | 0 | 0.418 | 0.330 | 0 | 0 | 14.70 | 0 | 14.74 | 0 | 0 | 0 | 0 |
| 490.00 | 84.00 | 15.87 | 0.00 | 0.310 | 0.000 | 0.000 | 0 | 0.253 | 0 | 0 | 1.080 | 1.334 | 0 | 0 | 26.68 | 0 | 148.68 | 0 | 0 | 0 | 0 |
| 544.00 | 82.50 | 19.57 | 0.00 | 0.675 | 0.000 | 0.000 | 0 | 0.231 | 0 | 0 | 0.560 | 1.434 | 0 | 0 | 33.80 | 0 | 142.00 | 0 | 0 | 0 | 0 |
| 670.00 | 75.00 | 23.30 | 0.00 | 0.133 | 0.000 | 0.000 | 0 | 0.173 | 0 | 0 | 1.681 | 1.024 | 0 | 0 | 58.02 | 0 | 167.40 | 0 | 0 | 0 | 0 |
| 700.00 | 75.60 | 30.30 | 0.00 | 0.358 | 0.000 | 0.000 | 0 | 0.201 | 0 | 0 | 1.380 | 0.917 | 0 | 0 | 54.23 | 0 | 253.20 | 0 | 0 | 0 | 0 |
| 440.00 | 38.00 | 2.30 | 0.00 | 0.266 | 0.000 | 0.000 | 0 | 0.150 | 0 | 0 | 0.443 | 1.600 | 0 | 0 | 11.55 | 0 | 2.38 | 0 | 0 | 0 | 0 |

**Table 2** Means of detectable elements in human lithogenic bile according to Source and Sex

| *Element* | *Mean, Values and Standard deviation µgm/ml* | | | | | |
|---|---|---|---|---|---|---|
| | *In Hepatic bile* | *In C.B.D. bile* | *In G.B. bile* | *Regardless of source* | *In Males* | *In Females* |
| Na | 335±89.1 | 479.76±125.85 | 523.22±137.77 | 506.77±136.11 | 507.33±96.06 | 506.55±150.18 |
| K | 48.4±8.48 | 50.84±16.19 | 73.56±25.07 | 65.52±24.58 | 73.92±26.74 | 62.27±23.34 |
| P | 23.70±23.05 | 37.116±44.07 | 141.345±92.66 | 104.36±92.96 | 151.24±100.4 | 86.21±84.73 |
| Ca | 16.47±2.54 | 17.25±8.2 | 44.29±25.19 | 34.82±24.47 | 44.51±26.91 | 31.07±22.81 |
| Mg | 5.22±3.78 | 5.30±3.36 | 16.76±9.57 | 12.77±9.64 | 17.04±10.71 | 11.11±8.84 |
| Mn | 0.145±0.08 | 0.1647±.0.133 | 0.304±0.211 | 0.254±0.196 | 0.236±0.161 | 0.261±0.208 |
| Pb | 0.125±0.176 | 0.258±0.301 | 0.365±0.303 | 0.322±0.300 | 0.409±0.210 | 0.288±0.325 |
| Fe | 0.440±0.065 | 0.475±0.099 | 1.105±0.545 | 0.884±0.536 | 0.936±0.711 | 0.864±0.465 |
| Ag | 0.025±0.035 | 00 | 0.040±0.087 | 0.028±0.072 | 0.048±0.071 | 0.019±0.073 |
| Cu | 0.883±0.418 | 0.669±0.574 | 0.949±0.531 | 0.862±0.545 | 1.098±0.654 | 0.770±0.477 |
| Zn | 1.099±0.799 | 0.306±0.204 | 0.298±0.531 | 0.338±0.265 | 0.281±0.137 | 0.360±0.299 |
| Ni | 000 | 000 | 0.0046±0.024 | 0.003±0.019 | 0.011±0.037 | 000 |

digestion method was applied in the hope off assessing the entire trace element content of different constituents of human bile; because using supernatant only[1] or including the gallbladder tissue[2] well either exclude from or add to, the bile itself HCl can form soluble salts with all the assessed elements[3]; it's ability to digest bile make it all the more attractive, if elements are present in the mucous secreted by the gallbladder wall[4] into the bile they will be extracted the HCl. We found that only 3 molar (3M) HCl can form a solution with bile; HCl of molarity less than 3M failed to form a solution and if molarity was more than 3M, it coagulated the bile. The reason for using 2 different machines is for maximum accuracy and sensitivity:[5] measurements are given as µg/ml and that is why comparing values with other studies are difficult. The final value given to an element is obtained after subtracting the content of the given element with HCl solvent from the sample value. Twenty-two elements were studied (Table 1) 2 elements were assessed for the first time (Ba & Si); out of 22 elements only 12 can be detected in human bile, the mean values of those elements in hepatic, CBD & gallbladder bile and also regardless the source are given (Table 2). Table 2 also shows the mean value of female and male bile regardless of the source.

Male and female bile contain these elements in almost equal amounts with perhaps slightly more in the male except for Zn where it is slightly more in females. Gallbladder bile content of detected elements is around 1.5–2 times greater than that in hepatic bile; in the case of (Mg & P) gallbladder bile is 3 and 6 times respectively, more than that of hepatic bile; Zn is exceptional in that, it is 3.6 times more in hepatic bile than gallbladder bile.

Gallbladder concentrates bile by absorbing water[6] and so the concentration of solutes will be increased; if water absorption is the cause of the higher concentration of elements in gallbladder bile then, one would expect universal and uniform increase of all solutes but this is not the case. The gallbladder mucosa has an absorptive capacity greater than that of small intestine per unit of surface, it can absorb and secrete electrolytes; the high concentration of Sodium Na, Potassium K and Calcium Ca, can be explained by water and electrolyte absorption from the gallbladder mucosa[7–11].

Copper levels are almost equal in hepatic and gallbladder bile and this can be explained by the fact that copper in the bile is attached to lignads and is not reabsorbable[12]. Only in the gallbladder (not in intrahepatic bile ducts) the supersaturated bile can form crystals which eventually may form stones; bile from gallbladders with stones have high concentration of glycoproteins which are also found in the gallbladder wall and gallbladder stones[13–15]. Excessive production of glycoproteins by gallbladder mucosa precedes stone formation[16], such glycoproteins are thought to be the organic matrix present in the center of gallstone which trap calcium compounds as calcium phosphate or bilirubinate to form the nidus of a stone around which crystals aggregate[17–20].

Not every supersaturated bile present in the gallbladder can form crystals the difference between supersaturated bile in forming crystals is in the nucleation capacity[21]. Nucleation is a feature of stone forming bile it depends only on the presence of supersaturated bile in the gallbladder, but is also dependent on the addition of a nucleating agent (e.g. glycoprotein)[22], lack of an agent in normal bile; or absence of an inhibitor of crystalization or combination. So, both cholesterol saturation and nucleating factor(s) or lack of inhibitor(s) must be present in gallbladder bile before a stone can be formed. The high levels of Mg, P, pb, Fe and Mn noticed in this study may be due to secretion of these metals from the gallbladder mucosa either in ionic or compound forms, this may be particularly true in the case of Ni as it is only found in gallbladder bile; some of these elements may play a role in forming the gallstone nidus. Mg is known to increase the solubility of Ca making it available to react with Phosphorus an ion present in abundance in gallbladder bile; as a result calcium phosphate can be formed and precipitated in the already present organic matrix formed by glycoproteins.

The exceptionally low level of Zn in gallbladder bile may be due to absorption of this metal from the gallbladder mucosa because such a low level is not due to methodological reasons; such observation was also noted previously[(1)].

Zn was found to be higher in non-lithogenic bile than abnormal bile[(1)]. The findings of one study however did not conclusively demonstrate a bile lipoprotein complex as an inhibitor of crystalization, it was not possible to exclude the possibility that there could be a naturally occurring nucleating-inhibiting factor which may be one of the bile solutes[(23)]. Observation of this study may indicate that Zn is probably acting as a protective element against formation of stones in gallbladder as in urinary stones[24] or a crystalization inhibitor but confirmation cannot be provided in this study due to absence of normal bile samples.

In conclusion, trace element concentration in gallbladder bile is more than in hepatic bile and this cannot be only due to water absorption; Zn is exceptionally low in gallbladder bile an observation which deserves further investigation as Zn may be a protective element against stone formation.

### ACKNOWLEDGEMENT

I would like to thank Dr. S. Bahaffi for his assistance and also to Ms. Buen V. Nolasco for scretarial work.

### REFERENCES

1. Harvey, R.C., Taylor, D., Petrunka, C.N., *et al.* (1985)Quantitative analysis of major, minor and trace elements in gallbladder bile of patients with and without gallstones. *Hepatology,* **5**, No.1, pp 129–132.
2. Markkanen, T., Aho, A.J. (1972 ) Metabolic aspects of trace metal content of gallstones and gallbladder. *Acta chir Scand,* **138**, 301–305.
3. Bassett, J., Denney, R.C., Jeffrey, G.H. In vogel's textbook of quantitative inorganic analysis. Fourth edition, Lougman, pp 296–297.
4. Mc, A.I., Mackay, C. In Jamiesan & Kay's textbook of surgical pathology. 3rd edition, churchill living stone, pp. 284.
5. Fifield, F.W., Kealey, D. In principles and practice of analytical chemistry. 2nd edition, International Textbook Company. pp 266–267.
6. Wheeler, H.O (1971). Concentrating function of the gallbladder. *Am J Med.,* **51**, 588–592.
7. Dietschy, J.M (1964). Water and solute movement across the wall of the everted rabbit gallbladder. *Gastroenterol,* **47**, 395–400.
8. Frizzel, R.A., Field, M., Schultz, S.G (1979). Sodium–coupled chloride transport by epithelial tissues. *Am J physiology,* **236**, F1.
9. Frizzel, R.A., Heintzuk (1980). Transport function of gallbladder. I*nt. Rev physiol,* **21**, 221–224.
10. Duffy, M.E., Turnheim, K, Frizzell, R.A., *et al.* (1978) Intracellular chloride activities in rabbit gallbladder, direct evidence for the role of the sodium-gradient in energizing "uphill" chloride transport. *J Membr. Biol,* **42**, 229–233.
11. Wood, J.R., Svanvik, J (1983). Gallbladder water and electrolyte transport and its regulation. *Gut,* **24**, 579–593.
12. McIntyre, N. Liver function in surgery of the liver and biliary system. 1st edition, churchill living stone, vol 1, pp 46.
13. Bouchier, A.D., Cooperband, S.R., El Kodsi, B.M (1965). Mucous substances and viscosity of normal and pathological human bile. *Gastroenterol,* **49**, 343–353.
14. Lee, S.P., Lim, T.H., Scott, A.J (1979). Carbohydrate Moieties glycoproteins in human hepatic and gallbladder bile, gallbladder mucosa and gallsones. *Clinical Science,* **56**, 533–538.
15. Womack, N.A (1971). The development of gallstones. *Surgery gynaecology and obstetrics,* **133**, 937–945.
16. Lee, S.P., La Mount, T., Carey, M.C. (1981).Role of gallbladder mucous hypersecretion in the evolution of cholesterol gallstones. *Journal of Clinical Investigation,* **67**, 1712–23.
17. Sutor, D.J., Wooley, S.E (1974). Organic matrix of gallstones. *Gut* **15**, 487–491.
18. Bills, P.M., Lewis, D (1975). A structural study of gallstones. *Gut* **16**, 630–637.
19. Beer, J.M., Bills, P.M., Lewis, D (1977). Electron probe microanalysis in the study of gallstones. *Gut,* **18**, 836–942.
20. Beer, J.M., Bills, P.M., Lewis, D (1979). Microstructure of gallstones. *Gastroenterol,* **76**, 548–555.
21. Holzbuch, R.T., Marsh, M.E., Olzewski, M., *et al.* (1973). Cholesterol solubility in bile: Evidence that Supersaturated Bile is Frequent in Healthy Man. *J Clin Invest,* **52**, 1467–79.
22. Burnstein, M.J., Ilson, R.G., Petrunka, C.N., *et al.* (1983). Evidence for a potent nucleating factor in the gallbladder bile of patients with cholesterol gallstones. *Gastroenterol,* **85**, 801–7.
23. Holzbach, R.T., Kibe, A., Thiel, E., *et al.* (1984). Biliary proteins unique inhibitors of cholesterol crystal nucleation in human gallbladder bile. *J Clin Invest,* **73**, 35–45.
24. Elliot, J.S., Eusebio, E. (1967). Calcium oxalate solubility. The Effect of Trace Metals. *J Invest Urol,* **9**, 428–433.

